# Factors Associated With Adherence to COVID-19 Preventive Measures Among Saudi Arabians

**DOI:** 10.7759/cureus.14623

**Published:** 2021-04-22

**Authors:** Abdullah S Alshammari, Hotoon Alshammari, Sulaiman Alshammari

**Affiliations:** 1 Medicine, College of Medicine, Imam Mohammad Ibn Saud Islamic University, Riyadh, Riyadh, SAU; 2 Family and Community Medicine, College of Medicine, AlMaarefa University, Riyadh, SAU; 3 Family and Community Medicine, College of Medicine, King Saud University, Riyadh, SAU; 4 Family and Community Medicine, The Health Promotion and Health Education Research Chair, King Saud University, Riyadh, SAU

**Keywords:** coronavirus, perception of susceptibility, seriousness, adherence to preventive measures, covid-19 pandemic

## Abstract

Background

A recent outbreak of COVID-19 which was initially reported in Wuhan City, China, has become a worldwide pandemic. This global public health threat drew the attention of the medical and scientific community to make the necessary research efforts to combat the spread of the virus. Predictors of preventive behaviors could be of considerable importance relevant to identifying high-risk groups to manage their behavior. We aimed to investigate the association between people's perception of COVID-19 adherence to preventive measures, susceptibility, seriousness, benefits, motivators, barriers, confidence, and information sources.

Methods

We conducted a cross-sectional study during the early phase of the COVID-19 pandemic, enrolling 1,568 participants from WhatsApp groups, aged 16 years and above from March to April 2020 in Saudi Arabia. We prepared an online Arabic self-administered questionnaire, which consisted of (1) sociodemographic characteristics; (2) compliance to preventive measures, including the perception of susceptibility, seriousness, benefits, motivation, barriers to preventive measures, self-confidence; and (3) sources of COVID-19 information.

Results

Of the 1,568 participants, 60% (n = 1,004) were women, 54.1% (n = 459) were unmarried, and 80.2% (n = 1,258) had earned university degrees. Regarding compliance and perception toward preventive measures, 64% of participants washed hands with soap and water; 50% followed the cough etiquette; 17.5% avoided touching their eyes, nose, and mouth; and 34.6% never shook hands with others. Approximately 52.2%-69.7% avoided going to crowded places and wore facial masks as needed. In addition, 24.3% of people observed social distancing. However, 52.4% did not receive the annual flu vaccine. Approximately 83% used banknotes, 69.8% made online payments, and 51.8% avoided ATM/credit card use. The perception of susceptibility and the seriousness of COVID-19 infection accounted for 8.5% and 50% of participants, respectively. Perceived benefits and motivation reached more than 90%. Barriers for not being able to practice preventive measures were the fear of nasal swabs, forgetfulness, and negligence, which rated 39% and 66%, respectively. Confidence in practicing the preventive measures and distinguishing the symptoms of COVID-19 accounted for 80% and 60% of the participants, respectively. The most used sources to retrieve information were the Ministry of Health (MOH) news and reports and social media, representing 53.5% and 24.6%, respectively.

Conclusions

During the early phase of the pandemic, people's perception of susceptibility was low, which resulted in a lack of awareness of some citizens to take the COVID-19 infection seriously. Thus, the benefits of preventive measures did not fully translate into practice. The current successful decline of COVID-19 cases should be accompanied by continuing health promotion and disease prevention programs utilizing all avenues and opportunities to remind people of scientific, religious, and cultural practices of handwashing, coughing etiquette. The persistent use of personal protective equipment (PPE), such as facial masks, gloves, and, for frontline workers, shields and protective garments is necessary. Family physicians and teams should play a vital role in this battle.

## Introduction

The recent COVID-19 infection outbreak has turned out to be a global public health catastrophe affecting all aspects of people's lives. Currently, national and international health agencies are working relentlessly to save our world from the shattering effects of COVID-19. Simultaneously, scientists in field worldwide are making the necessary efforts to find the mechanism of transmission, clinical aspects, diagnostic tools, prevention, and treatment interventions. The World Health Organization (WHO) has documented more than 20 infectious agents over the past two decades, including COVID-19 [1]. The COVID-19 outbreak started in Wuhan, China, on December 31, 2019 [2]. Later in January 30, 2020, the WHO Emergency Committee declared that the outbreak represents “a Public Health Emergency of International Concern.” By March 11, 2020, the WHO declared COVID-19 as a pandemic [3]. The coronavirus infection has spread rapidly worldwide, claiming so many lives. As of May 5, 2020, 3,600,1063 people had contracted COVID-19, with 251,898 deaths in 212 countries [4]. The disease has transmitted to countries and territories worldwide, and as of September 24, 2020, these figures had jumped to 32,330,254 cases and 985,585 deaths in 215 counties.

Since the announcement of the first case of COVID-19 in Saudi Arabia on March 3, 2020, 331,857 have contracted the virus, with 4,599 deaths [5]. The person-to-person transmission of the virus mainly occurs via respiratory droplets, close contact of healthy persons with infected individuals (within approximately six feet) during talking, sneezing, coughing, and indirect contact with contaminated objects or surfaces [6]. COVID-19 is a highly contagious disease with an average number of 2-3.5 new infections caused by an infected person in an uninfected population [7]. The disease spectrum of COVID-19 varies from asymptomatic carriers to life-threatening conditions [8]. Symptomatic people are most contagious. During the viral incubation period (approximately 5.5 days), asymptomatic carriers are a major source of infection [9]. Clinical and laboratory manifestations include fever, dry cough, dyspnea, myalgia, fatigue, lymphopenia, and pneumonia. Among the most frequently reported complications of the COVID-19 infection are arrhythmia, renal injury, acute cardiac injury, acute respiratory distress syndrome (ARDS), and shock [10]. The critically ill patients aged >65 years with chronic medical conditions are a high-risk group of mortality [11]. The fast-spreading nature of the pandemic threatened to overwhelm healthcare systems and the economy worldwide [12]. People may need to live with social distancing for a long time to prevent thousands from dying. Aggressive testing, coupled with relentless contact tracing, quarantine of those exposed to the infection, and isolation of the sick are strategies to control the disease's spread. Currently, there is no approved pharmacological treatment for COVID-19 infections. At this point, supportive and preventive measures remain the best weapon. Nevertheless, supportive medical care, such as the use of air pump ventilators to aid breathing and dialysis to aid failing kidneys, is used frequently in critical cases to save lives of patients with COVID-19 [13]. Proven preventive procedures are good hygiene, antiseptic use, and social distancing. The WHO recommends the following measures to prevent COVID-19 transmission [14]: (1) frequently clean hands with soap and water, or antiseptics; (2) practice social distancing; (3) avoid touching eyes, nose, or mouth; (4) practice safe cough etiquette; (5) stay home; (6) if symptoms develop, seek medical care; (7) adhere to the directions of a health authority; (8) restrict unnecessary visits to healthcare facilities to avoid acquiring COVID-19 infection; and (9) permit the systems to function efficiently.

Regular monitoring of knowledge via updates of the disease, attitude, perceptions, and practice toward the outbreak is essential to highlight the areas for improvement and interventions to prevent COVID-19, particularly as a baseline at the start of the epidemic. As we learn more about the pandemic, we may be more precise in fighting it, finding a balance between protecting people, allowing the economy to restore, and returning to society functions again. Thereby, the health behavior of the public is vital in the prevention of respiratory infections, such as COVD-19. Thus, this study investigated the association between people's perception of susceptibility, the seriousness of COVID-19 infection, benefits, motivation, barriers, confidence, and adherence with preventive measures. Additionally, we investigated the public source of health information relative to the disease.

## Materials and methods

We conducted this cross-sectional study during March 23-April 4, 2020, in the early phase of the COVID-19 pandemic, utilizing WhatsApp groups in Saudi Arabia. The inclusion criteria were persons older than 18 years, not diagnosed with COVID-19, Arabic speakers, and who gave written consent to participate in the study. The investigators prepared Arabic predesigned self-administered questionnaire with 20 questions, which had four main parts: (1) Sociodemographic, including age, sex, employment status, and educational level. (2) Assessment of the degree of public compliance with and practice of preventive measures against COVID-19 infections using a five-point Likert scale (always = 5, often = 4, sometimes = 3, rarely = 2, and never = 1) for scoring. We categorized the total score of the 20 questions on practice to prevent COVID-19 infections into good (median and above) and bad (below the median score) practices. (3) We used 30 statements to assess the participants' health beliefs about perceived susceptibility, seriousness, benefits, motivation, barriers, and self-confidence about COVID-19 and a five-point Likert-type scale for options (strongly disagree [one point] to strongly agree [five points]). We designed and modified these parts according to the literature [15,16]. We assessed each subscale separately and calculated the total score for each participant. Higher scores indicated stronger feelings about that construct. (4) We also queried the population's source of information about the prevention of respiratory infections.

Five academic staff members (two family physicians, one infectious disease consultant, one community professor, and one biostatistician) reviewed the study's instrument. Before the main study, we conducted a pilot questionnaire study with 50 individuals to check the applicability and clarity, cultural and scientific appropriateness, identification of any difficulties with the questionnaire, and estimated the time required to complete the questionnaire. The questionnaires took approximately 10-15 minutes to complete. The authors excluded participants in the pilot study from the main study. The internal consistency measure (Cronbach's alpha) of the modified instrument was 0.86.

The Institutional Review Board of College of Medicine, King Saud University approved this study (Research Project No. E-20-5101). All participants agreed to take part through a "check sign" in the informed written consent. The participants provided their information strictly anonymously and confidential. We analyzed the data using SPSS 21.0 software (IBM Corp., Armonk, NY). We reported the sociodemographic characteristics, preventive measures against COVID-19, and health belief domains (HBM) scale as frequency, percentage, and mean as appropriate. We used median estimations to categorize preventive measures practice as good and poor. P values < 0.05 denoted a statistically significant difference between compared variables.

## Results

Table 1 shows that 1,568 individuals participated in the current study. Those aged under 18 years represented approximately 5% of the participants. Moreover, approximately 80% were between the ages of 18 and 54 years, and 15% were aged above 55 years. The majority of participants were females, accounting for 60%. The number of unmarried individuals was approximately 54.1%. The majority (80.2%) of the participants had a university degree or higher. Those who are currently not working accounted for 56.8%.

**Table 1:** Demographic characteristics of the participants (N = 1568)

Variables	Frequency	Percentage
Age (years)		
Under 18	77	4.9
18–34	838	53.4
35–54	425	27.1
55+	228	14.5
Sex		
Male	601	38.3
Female	967	61.7
Marital status		
Not married	848	54.1
Married	720	45.9
Educational level		
Below secondary	31	2
Secondary school	280	17.9
University	1257	80.2
Occupation		
Not working	890	56.8
Working	423	27
Health professionals	255	16.3
Total	1568	100

Sixty-four percent of the participants washed their hands with water and soap, with approximately 60.9% washing before eating and 74.2% on entering their home (Table 2). Furthermore, 47.0% removed their shoes on entering their home, and at the same time, antiseptic soap for handwashing accounted for 31.8% on surfaces and 39.0% on hands. Approximately 50% always followed the preventive instructions for coughing and sneezing while using a tissue or elbow. Those who avoided touching eyes, nose, and mouth directly accounted for 17.5%, and 34.6% avoided shaking hands with others. Moreover, 80.1% never shared their personal items, such as towels and reusable napkins with others. People who took precautionary measures by avoiding crowded places and wore masks as needed were between 52.2% and 69.7%. People who complied with the recommended standard distance when communicating with others accounted for 24.3%. Those who never took the annual flu vaccine accounted for approximately 52.4%. When it came to handling money, most participants (82.8%) still used banknotes, 51.8% avoided ATM/credit cards, and 69.8% used online payment options.

**Table 2:** Practice of preventive measure against COVID-19 by Likert scale (N = 1568)

Questions	Always (%)	Often (%)	Sometimes (%)	Never (%)	
					Do you wash your hand with water and soap constantly?	1003 (64.0)	440 (28.1)	123 (7.8)	2 (0.1)	
Do you wash your hands with water and soap before eating?	955 (60.9)	358 (22.8)	215 (13.7)	40 (2.6)	
Are you keen on using antiseptic soap for hands?	455 (29.0)	498 (31.8)	477 (30.4)	138 (8.8)	
Are you keen on using antiseptic soap for surfaces, such as tables and floor?	498 (31.8)	478 (30.5)	435 (27.7)	157 (10.0)	
Do you avoid touching your eyes, nose, and mouth directly with your hands?	274 (17.5)	602 (38.4)	533 (34.0)	159 (10.1)	
Do you cover your nose and mouth while sneezing or coughing with tissue and throw it in the garbage?	789 (50.3)	445 (28.4)	260 (16.6)	74 (4.7)	
In case you do not have tissue, do you use your elbow while sneezing or coughing?	725 (46.2)	415 (26.5)	271 (17.3)	157 (10.0)	
Do you share your personal belongings with others (e.g., towels)?	42 (2.7)	63 (4.0)	191 (12.2)	1272 (81.1)	
					Do you avoid crowded places?	818 (52.2)	450 (28.7)	225 (14.3)	75 (4.8)	
Do you wear mask in crowded places?	599 (38.2)	386 (18.2)	298 (19.0)	385 (24.6)	
Do you avoid people with respiratory infection?	957 (61.0)	326 (20.8)	181 (11.5)	104 (6.6)	
In case you are around infected people, do you wear mask?	1093 (69.7)	190 (12.1)	138 (8.8)	147 (9.4)	
Do you take the seasonal flu vaccine?	271 (17.3)	177 (11.3)	298 (19.0)	822 (52.4)	
Do you shake hands with others?	248 (15.8)	330 (21.0)	447 (28.5)	543 (34.6)	
Do you avoid using banknotes in buying and selling?	348 (22.2)	440 (28.1)	510 (32.5)	270 (17.2)	
Do you avoid using ATM and credit cards in buying and selling?	190 (12.1)	225 (14.2)	398 (25.4)	755 (48.2)	
Do you use an online payment method, such as Apple Pay, STC pay, Google Pay etc.?	467 (29.8)	357 (22.8)	271 (17.3)	473 (30.2)	
Do you take your shoes off when entering the house?	737 (47.0)	255 (16.3)	256 (16.3)	320 (20.4)	
Do you stand at least a meter away while communicating with others?	384 (24.5)	514 (32.8)	506 (32.3)	164 (10.5)	
Do you wash your hand with water and soap when entering the house?	1163 (74.2)	248 (15.8)	119 (7.6)	38 (2.4)	

When comparing preventive practices, there were no significant differences between the age groups (Table 3). The age group of 18-34 (54.1%) years had the best preventive practice than other age groups. The preventive practice among females (59.6%) was significantly better than in males (50.1%). Married participants (57.1%) had better preventive practice than those in the unmarried group (55.0%). The preventive practices among participants with different levels of education were not statistically different.

**Table 3:** COVID-19 preventive practice measures

Variable	Preventive Practice Mean		Total (%)	P Value
	Poor Practice (%)	Good Practice (%)		
				Age (years)				
Under 18	32 (41.6)	45 (58.4)	77 (100.0)	0.45
18–34	385 (45.9)	453 (54.1)	838 (100.0)	
35–54	177 (41.6)	248 (58.4)	425 (100.0)	
55+	97 (42.5)	131 (57.5)	228 (100.0)	
Sex				
Male	300 (49.9)	301 (50.1)	601 (100.0)	
Female	391 (40.4)	576 (59.6)	967 (100.0)	0.0001
Marital status				
Not married	382 (45.0)	466 (55.0)	848 (100.0)	
Married	309 (42.9)	411 (57.1)	720 (100.0)	0.21
Educational level				
Below secondary	12 (38.7)	19 (61.3)	31 (100.0)	
Secondary school	131 (46.8)	149 (53.2)	280 (100.0)	
University	548 (43.6)	709 (56.4)	1257 (100.0)	0.51
Occupation				
Not working	410 (46.1)	480 (53.9)	890 (100.0)	
Working	193 (45.6)	230 (54.4)	423 (100.0)	
Health professionals	88 (34.5)	167 (65.5)	255 (100.0)	
Total	691 (44.1)	877 (55.9)	1568 (100.0)	0.003

Table 4 displays the frequency of various domains of health beliefs of the participants. On the susceptible side, approximately 8.5% felt they were susceptible to COVID-19 infection. Approximately 50% of participants believed that COVID-19 infection was a scaring condition and were worried about getting symptoms that would threaten their relationship with relatives. More than half of the participants were not concerned about losing their incomes and lives. Between 73.4% and 95.0% of participants perceived the benefits of preventive measures as reduced chances of getting infected, being admitted to the hospital, and losing their lives.

**Table 4:** Frequency of various domains of health beliefs of the participants (N = 1568)

Variables	Agree	Neutral	Disagree
	N (%)	N (%)	N (%)
Susceptibility			
It is likely that I will get COVID-19 in these days.	133 (8.5)	562 (35.8)	873 (55.7)
My chance of getting COVID-19 is higher than the others.	142 (9.1)	519 (33.1)	907 (57.8)
My family’s chances of getting COVID-19 is high.	205 (13.1)	440 (28.1)	923 (58.9)
Seriousness			
The thought of COVID-19 scares me.	714 (45.5)	349 (22.3)	505 (32.2)
When I think about COVID-19, my heart beats faster.	581 (37.1)	332 (21.2)	655 (41.8)
If I get infected with COVID-19, I will get the symptoms.	766 (48.9)	525 (33.5)	277 (17.7)
If I get infected with COVID-19, I will lose my income.	200 (12.8)	328 (20.9)	1040 (66.3)
COVID-19 infection would threaten my relationship with relatives.	846 (54.0)	268 (17.1)	454 (29.0)
If I get infected with COVID-19, I will not live long.	134 (8.5)	460 (29.3)	974 (62.1)
Benefits			
I feel satisfied when I do the preventive measures against COVID-19, such as hand washing.	1489 (95.0)	54 (3.4)	25 (1.6)
I do not worry about getting infected with COVID-19 when I practice the preventive measures.	1151 (73.4)	278 (17.7)	139 (8.9)
Practicing the preventive measures against COVID-19 will reduce the likelihood of death from infection.	1330 (84.8)	180 (11.5)	58 (3.7)
Practicing the preventive measures against COVID-19 will reduce the likelihood of hospital admission.	1395 (89.0)	130 (8.3)	43 (2.7)
Motivation			
Maintaining healthy well-being is a top priority for me.	1508 (96.2)	45 (2.9)	15 (1.0)
I feel it is important to perform physical activity to improve my health.	1456 (92.9)	94 (6.0)	18 (1.1)
I eat well-balanced meals for my health.	1135 (72.4)	283 (18.0)	150 (9.6)
I have regular health checkups even when I am not sick.	596 (38.0)	403 (25.7)	569 (36.3)
I exercise at least three times a week for my health.	693 (44.2)	345 (22.0)	530 (33.8)
I seek new health information to promote my health.	1202 (76.7)	249 (15.9)	117 (7.5)
Barriers			
I am afraid to have a nasal swab.	611 (39.0)	515 (32.8)	442 (28.2)
I am afraid to use antiseptic because of adverse effects, such as skin irritation and allergy.	397 (25.3)	281 (17.9)	890 (56.8)
It is difficult to get personal protective equipment (PPE) such as masks, antiseptics, detergents, etc.	604 (38.5)	257 (16.4)	707 (45.1)
I feel embarrassed when refusing shaking hands or hugging friends.	469 (29.9)	233 (14.9)	866 (55.2)
Forgetfulness or negligence is the cause for not practicing the preventive measures against COVID-19 infection.	1036 (66.1)	267 (17.0)	265 (16.9)
The cost of personal protective equipment's (PPE) is the cause for not practicing the preventive measures against COVID-19 infection.	380 (24.2)	352 (22.4)	836 (53.3)
Confidence			
I know how to practice the protective measures against COVID-19 infection.	1314 (83.8)	186 (11.9)	68 (4.3)
I am confident to practice the protective measures against COVID-19 infection correctly.	1279 (81.6)	232 (14.8)	57 (3.6)
I will detect COVID-19 infection if the symptoms appear.	975 (62.2)	142 (26.3)	181 (11.5)
I am able to differentiate between COVID-19 and common cold symptoms.	911 (58.1)	360 (23.0)	297 (18.9)
I am confident about the procedures I should follow when the symptoms appear or when I come in contact with infected people.	1261 (80.4)	208 (13.3)	99 (6.3)

The range of motivation varied. Between 38.0% and 96.2% of participants were motivated to maintain their healthy well-being by performing physical activity, exercising three times a week, eating balanced meals, having regular checkups, and keeping themselves updated for new health information.

Regarding perceived barriers to performing preventive measures, between 39% and 66% attributed their barriers to fear of undergoing nasal swab or forgetfulness or negligence in performing preventive measures. However, approximately 50% of participants did not feel that the availability or cost of personal protective equipment (PPE), side effect of antiseptic use, and avoiding the cultural handshaking and hugging relatives or friends were barriers to following the preventive measures against COVID-19 infection.

More than 80% of the participants were confident about performing the preventive measures correctly, and approximately 60% were aware of the signs and symptoms of being infected with COVID-19 and could distinguish the condition from common cold symptoms.

People perceived themselves as being susceptible to COVID-19 infection as they aged, by being male sex, by being married, and being health workers; and these were statistically significant (Table 5). Individuals of young age, women, those with low education, and those unemployed had a higher perception of the disease's seriousness than their counterparts. The groups with young age and low education perceived prevention benefits more than their counterparts. Men and married individuals perceived more barriers to performing preventive measures than others; these were statistically significant. The older participants, men, married individuals, those with an education level higher than secondary school, and health professionals were more motivated toward disease prevention. Even though these latter variables were statistically significant findings, the differences between the means were marginal (decimal differences).

**Table 5:** Demographic characteristics versus health belief model (N = 1568) *** denotes statistical significance.

Variable	N	Susceptibility	Seriousness	Benefits	Barriers	Motivation	Confidence
		(Mean + SD)	(Mean + SD)	(Mean + SD)	(Mean + SD)	(Mean + SD)	(Mean + SD)
Age (years)		***	***	***		***	***
Under 18	77	6.6 (2.8)	18.5 (5.0)	17.8 (2.2)	17.1 (4.8)	22.4 (4.5)	20.7 (3.7)
18–34	838	6.7 (2.7)	17.8 (4.9)	17.0 (2.4)	17.4 (4.4)	22.6 (4.0)	19.2 (3.7)
35–54	425	7.0 (2.6)	17.1 (4.6)	17.2 (2.2)	17.7 (4.1)	24.1 (3.5)	19.6 (3.3)
55+	228	7.1 (2.6)	16.4 (4.4)	17.3 (2.1)	17.7 (4.2)	25.1 (3.1)	19.9 (2.9)
Sex		***	***		***	***	
Male	601	7.2 (2.6)	17.0 (4.9)	17.2 (2.3)	18.1 (4.5)	23.9 (3.8)	19.5 (3.3)
Female	967	6.6 (2.6)	17.7 (4.8)	17.1 (2.3)	17.2 (4.2)	23.0 (3.9)	19.5 (3.6)
Marital status		***			***	***	
Not married	848	6.7 (2.6)	17.2 (4.7)	17.2 (2.2)	17.9 (4.2)	24.2 (3.5)	19.6 (3.3)
Married	720	7.0 (2.7)	17.9 (5.4)	16.9 (3.1)	19.3 (5.6)	24.5 (4.7)	20.1 (4.3)
Educational level			***	***	***	***	
Below secondary	31	6.3 (3.5)	18.1 (5.0)	17.5 (2.4)	17.8 (4.6)	22.9 (4.3)	19.7 (3.8)
Secondary school	280	6.7 (2.7)	17.3 (4.7)	17.1 (2.2)	17.4 (4.2)	23.4 (3.7)	19.4 (3.4)
University	1257	6.9 (2.6)	17.5 (4.8)	17.2 (2.3)	17.8 (4.3)	23.2 (4.0)	19.4 (3.6)
Occupation			***		***	***	***
Not working	890	6.5 (2.6)	17.8 (4.8)	17.1 (2.3)	17.4 (4.3)	23.2 (3.8)	19.1 (3.3)
Working	423	6.8 (2.5)	16.7 (4.7)	17.0 (2.2)	17.0 (4.3)	24.0 (3.6)	20.4 (3.0)
Health professionals	255	7.9 (2.7)	17.4 (4.8)	17.1 (2.3)	17.5 (4.3)	23.3 (3.9)	19.5 (3.5)
Total	1568	6.8 (2.6)	17.4 (4.8)	17.1 (2.3)	17.5 (4.3)	23.3 (3.9)	19.5 (3.5)

Sources of information about participants by their demographic characteristics are given in Table 6. About 97% of our participants claimed they had some health information from different sources. Most of the participants (53.5%) received health information from the Ministry of Health (MOH) sources. More than 24.6% of the participants received some of the information through social media, and there were no differences among their demographic characteristics. Furthermore, health workers' educational efforts accounted for less than 2%.

**Table 6:** Sources of information about participants by their demographic characteristics (N = 1568) WHO, World Health Organization; MOH, Ministry of Health.

Variable	Sources of information							Total
	No information	WHO	MOH	Social Media	Media	Health Workers	Friends	1547
	47	194	828	381	54	24	19	
	-3.00%	-12.50%	-53.50%	-24.60%	-3.50%	-1.60%	-1.20%	
Age (years)								
Under 18	6 (7.9)	9 (11.8)	42 (55.3)	1418.4	2 (2.6)	00.0)	3 (3.9)	76
18–34	30 (3.6)	107 (12.9)	460 (55.6)	203 (24.5)	6 (0.7)	15 (1.8)	7 (0.8)	828
35–54	9 (2.1)	46 (11.0)	211 (50.4)	112 (26.7)	28 (6.7)	6 (1.4)	7 (1.7)	419
55+	2 (0.9)	32 (14.3)	115 (51.3)	52 (23.2)	18 (8.0)	3 (1.3)	2 (0.9)	224
sex								
Male	22 (3.7)	85 (14.5)	297 (50.5)	141 (24.0)	26 (4.4)	13 (2.2)	40.7	588
Female	25 (2.6)	109 (11.4)	531 (55.4)	240 (25.0)	28 (2.9)	11 (1.1)	15 (1.6)	959
Marital status								
Not married	30 (3.6)	107 (12.7)	471 (56.0)	203 (24.1)	7 (0.8)	11 (1.3)	12 (1.4)	841
Married	17 (2.4)	87 (12.3)	357 (50.6)	178 (25.2)	47 (6.7)	13 (1.8)	7 (1.0)	706
Educational level								
Below secondary	3 (9.7)	3 (9.7)	16 (51.6)	3 (9.7)	3 (9.7)	2 (6.5)	1 (3.2)	31
Secondary school	11 (4.0)	24 (8.7)	153 (55.2)	67 (24.2)	13 (4.7)	2 (0.7)	7 (2.5)	277
University	33 (2.7)	167 (13.5)	659 (53.2)	311 (25.1)	38 (3.1)	20 (1.6)	11 (0.9)	1239
education								
Not working	33 (3.8)	91 (10.3)	455 (51.7)	238 (27.0)	36 (4.1)	14 (1.6)	13 (1.5)	880
Working	9 (2.2)	41 (9.9)	235 (56.8)	109 (26.3)	14 (3.4)	1 (0.2)	5 (1.2)	414
Health professionals	5 (2.0)	62 (24.5)	138 (54.5)	34 (13.4)	4 (1.6)	9 (3.6)	1 (0.4)	253

## Discussion

We conducted this earlier study after the first confirmed cases of COVID-19 in Saudi Arabia. The government implemented a lockdown of most public and private services, and population movement restrictions nationwide, including Hajj and Umrah. Additionally, they closed the schools, as 107 countries did under the assumption that kids would be high community transmitters [17].

Implementing preventive measures in the early phase of the pandemic varied among countries, including the European countries. Italy applied the most stringent lockdown measures and experienced a tremendous COVID-19 burden in Europe [18].

The majority of the 1,568 participants were young or middle-aged and mainly women with university degrees or higher. Additionally, in a previous study, women showed over-representation in an online health survey [19]. They were usually keen on protecting themselves and their families' health than men. Our participants perceived a low susceptibility of 8.5% to COVID-19 infection, which conformed to a previous study on Middle East respiratory syndrome-related coronavirus (MERS-CoV) [15]. The perception of susceptibility to infection was associated with age, being male, married, and being a health worker, which were statistically significant.

Approximately half of the participants perceived COVID-19 infection as a severe condition. This perception was associated with younger participants, women, those with low education, and the unemployed. However, our findings on susceptibility and seriousness were less than that of a previous study in Hong Kong, where perceived susceptibility and severity were 89% and 97%, respectively. The Health Believe Model proved that perceived susceptibility led to behavior change [20], such as hand washing and social distancing behaviors as well as health promotion through food and water distribution. On the contrary, the misperception of COVID-19 infection risks being low led individuals to nonadherence to protective actions [21]. A study comparing Hong Kong and the United Kingdom reported that those who perceived disease severity as "high" were more likely to adopt social distancing. Those who perceived transmission as "easy"' were prone to general social distancing and contact avoidance [22]. Despite the low perception of susceptibility and seriousness of COVID-19, the majority of our participants (73.4%-95.0%) perceived the benefits of preventive measures in reducing odds of infection, hospital admission, and losing their lives. As expected, the fear of nasal swab or forgetfulness and negligence were significant barriers (39%-66%) regarding compliance with our participants toward preventive measures. However, about half of them did not feel that the availability or the costs of PPE, the side effect of antiseptic use, or avoiding hugging friends were barriers to following the preventive measures against COVID-19 infection. The majority of participants felt confident about practicing the preventive measures accurately (80%), were aware of the symptoms of COVID-19, and could distinguish those symptoms from common cold symptoms (60%). This finding was similar to that in a previous systematic review and meta-analysis [23]. The participants' motivation to promote health varied between 38.0% and 96.2%, and it had an association with older participants, being male, being married, having above secondary school educational level, and being health professionals. The findings showed that 60.9% and 74.2% of the participants always washed their hands with soap and water and adopted religious teaching of frequent hand washings, respectively-more importantly, after using the lavatory and before eating. The Islamic faith already encourages Muslims to follow preventive measures and treatment whenever available. Prophet Mohammed (peace be upon Him) consistently urged Muslims to wash their hands before and after meals, after using the toilet, after touching a cadaver or one's shoes, and taking care of anything suspected of being unclean in any capacity [24].

Furthermore, 47.0% of participants removed their shoes when entering their homes and at the same time used antiseptic soap for hand wash (39.0%) and to clean surfaces (31.8%). Approximately 50% always followed the preventive instructions for coughing and sneezing while using a tissue or elbow. Those who avoided touching their eyes, nose, and mouth accounted for 17.5%, and 34.6% of them avoided shaking hands with others. Moreover, 80.1% never shared their personal belongings, such as towels or reusable napkins. These findings provide a warning to improve suboptimal coherence to government implemented protective measures. Between 52.2% and 69.7% of participants took precautionary measures by avoiding crowded places and wore facial masks as needed. People who had a noncompliance attitude toward the recommended standard social distancing when communicating with others accounted for 24.3%. Our findings were probably less than Hong Kong but better than the United Kingdom, with regard to adopting social distancing among the citizens (Hong Kong: 32.4%-93.7%; United Kingdom: 17.6%-59.0%) and mask-wearing (Hong Kong: 98.8%; United Kingdom: 3.1%) [22]. When compared with preventive practices, no significant differences were found between the age groups. The preventive practice among females (59.6%) was higher than that among males (50.1%) and was highly statistically significant, which was consistent with previous studies [25]. Such findings stress the need for selective health promotion efforts. Additionally, and understandably, married participants (57.1%) had better preventive practice than unmarried participants (55.0%), as they are responsible for other family members. The poor attitude toward the annual flu vaccine was 52.4%, which emphasized the importance of conveying appropriate messages to the public to better understand the vaccine's benefits and improve its uptake. When it came to handling money, most participants (82.8%) still used banknotes, 51.8% avoided ATM/credit cards, and 69.8% used online payment as ways of preventing infection.

A substantial number of participants were not worried about losing their income, as reported from an economically disadvantaged or poor population, whereby the government secured their income by continuing payment of their monthly salary. Workers with unpaid sick leave were more prone to continue to work even when they were ill; therefore, they presented late to healthcare services. An individual's economic status and educational attainment, and specifically women, had adequate knowledge about COVID-19; thus, they were more likely to practice appropriate practices against the infection [26]. This association between knowledge and practices demonstrated the importance of prompt and accurate public health communication. Useful evidence from behavioral science for fighting the COVID-19 outbreak reported that effective behavioral interventions promoting practical individual actions could reduce the adverse psychological effects of isolation [27]. Approximately 97% of our participants claimed that they had some health information from different sources. However, most of the participants (53.5%) received health information from MOH sources, more than 24.6% received some information through social media, and there were no differences among their demographic characteristics. Furthermore, health workers' educational efforts accounted for less than 2%.

In the current COVID‐19 pandemic, responsibly and appropriately used social media and websites have the potential to disseminate crucial information rapidly and effectively. A Google search reported the information-seeking behavior of increasing awareness and public engagement on Facebook regarding the use of facial masks, handwashing, and the use of hand sanitizers, advertising brands, and demonstrating proper handwashing steps [28,29].

Additionally, authorities recently initiated a public awareness campaign on emerging COVID-19. Saudi Arabia's government and private sector collaborated to develop and launch 19 Apps and platforms that served public health functions and provided healthcare services. The Saudi Vision 2030 framework released in 2017 has paved the way for the digital transformation. Education processes continued using an established electronic learning infrastructure. They also used social media, websites, and short message service (SMS) text messages to improve awareness and uptake of preventive measures to reduce the risk of transmission [30].

Further, 16.3% of the participants were healthcare professionals, of which 65.5% were found to have good disease preventive practice than other groups. In a systematic review and meta-analysis, healthcare workers (HCWs) and medical students prioritized practicing hand hygiene to prevent COVID-19 (81.7%), but wearing a face mask to avoid COVID-19 transmission was suboptimal (73.4%) [23]. Further, a big part of keeping the healthcare system working well ensures that its workers remain healthy. A mandatory infection control course is required for all staff.

It is also possible that this pandemic will become endemic and stay with us in the coming years. While waiting for efficient and safe pharmacological interventions, proven preventive tools available to people are good hygiene, antiseptic use, and social distancing. Furthermore, individuals and community compliance with these measures will define how long a country will be in a risky zone before gradually reaching the new normal. However, governments could impose various measures, such as arresting those violating curfew and social distancing measures. Nevertheless, punishment should be the last resort, and their use also highlights the need for further education of population to break the mistrust between the people and received information.

Despite the noncompliance of a subgroup of the population, people understand how to keep themselves and others safe from the virus through preventive behavior [26]. We believe that the uptake of preventive measures after seven months of reinforcement of public health regulations is high. In addition to border control and social distancing, high-volume testing for COVID-19, aggressive contact tracing, and quarantine centers likely contributed substantially to the decline of COVID-19 cases. The adoption of virtual learning prevented disruptive interventions in schools [1]. Therefore, investigating and monitoring the disease from a holistic perspective, including the people's perception and behavior toward the COVID-19 infections, are necessary to allow healthcare providers, managers, and policymakers to improve an immediate response performance to the current and future pandemics. Dependable and well-timed health information is a crucial evidence-based approach for policy development and good health management. Progressive success in the decline of COVID-19 cases should emphasize promoting health and awareness, utilizing all means and media. We should seize every opportunity to remind people of religious and cultural practices of hand washing and covering noses and mouths, especially when sneezing and coughing. Furthermore, we strongly encourage the government and the public to invest in public health education, particularly, in schools and universities.

Limitations of this study

The people who participated in the study were selected from WhatsApp groups who had mostly higher level of education. Thus, we cannot generalize the findings to all populations. Additionally, we could not rule out the recall bias.

## Conclusions

During the pandemic's early days, there was a low perception of susceptibility, moderate perception of seriousness, high perception of benefits and motivation, and low perception of barriers among people toward COVID-19. However, not all those who perceived the benefits translated into the practice of preventive measures. Therefore, health officials should consider baseline levels of risk perception and knowledge in the populations as well as prior sensitization to infectious disease outbreaks during the development of mitigation strategies. Progressive success in the decline of COVID-19 cases should be emphasized persistently to promote health and awareness using the media and other means. We should seize every opportunity to remind people of religious and cultural practices of handwashing, covering noses, and mouths, especially when sneezing and coughing. Thus, primary healthcare physicians and teams should provide more educational information.
